# Role of simian virus 40 in cancer incidence in solid organ transplant patients

**DOI:** 10.1038/sj.bjc.6603107

**Published:** 2006-04-18

**Authors:** V Paracchini, A Nanni Costa, S Garte, E Taioli

**Affiliations:** 1 Department of Epidemiology, University of Pittsburgh Cancer Institute, 5150 Centre Avenue, Fourth Floor, Pittsburgh PA, USA; 2 Italian National Transplant Center, Istituto Superiore di Sanita, Viale Regina Elena 299, 00161 Roma, Italy; 3 Genetics Research Institute ONLUS, Strada della Carità 10, 20135 Milano, Italy

**Keywords:** epidemiology, models of viral transmission, cohort study

## Abstract

Transplant recipients have an increased risk of developing cancer in comparison with the general population. We present here data on cancer development in transplanted subjects who received organs from donors whose DNA was previously examined for the genomic insertion of Simian Virus 40 (SV40). Active follow-up of 387 recipients of solid organs donated by 134 donors, not clinically affected by cancer, was performed through the National Transplant Center (NTC). The average length of follow-up after transplant was 671±219 days (range 0–1085 days). Out of 134 proposed donors, 120 were utilised for organ donation. Of these, 12 (10%) were classified as positive for SV40 genomic insertion. None of the 41 recipients of organs from SV40 positive donors developed a tumour during the follow-up. In all, 11 recipients of organs given by SV40 negative donors developed a tumour (cancer incidence: 0.015 per year). In conclusion, cancer rates observed in our study are comparable to what reported by the literature in transplanted patients. Recipients of solid organs from SV40 positive donors do not have an increased risk of cancer after transplant. The role of SV40 in carcinogenesis in transplanted patients may be minimal.

One of the major consequences of solid organ transplantation is the high incidence of cancer after transplant. Transplant recipients have a three- to four-fold increased risk of developing cancer in comparison with the general population (Pedotti *et al*, 2003), at least for kidney and heart (Cardillo *et al*, 2001) transplants. Cancer occurrence in these patients has been attributed to the type and dose of immunosuppressive regimen, the presence of cancer in the donor's organ and to viral infections.

Simian virus 40 (SV40), a member of the polyomavirus family together with BK and JC, has been shown to be oncogenic in rodents, and has been detected in human tumours and tissues at variable frequencies. A systematic review of the literature indicates that the frequency of SV40 genomic infection in healthy subjects varies from 6 to 11% (Paracchini *et al*, 2006). The virus was inadvertently transmitted to humans through the Poliovirus vaccine in US between 1955 and 1963 (Butel and Lednicky, 1999a). However, epidemiological studies do not indicate an increased incidence of cancer associated with such contamination (Strickler *et al*, 2003). We have reported (Paracchini *et al*, 2005) that the prevalence of SV40 genomic infection in healthy subjects is not associated with year of birth, thus suggesting that factors other than polio vaccine could be responsible for the infection.

The role of SV40 sequences in human tumorigenesis remains controversial; polyomavirus infections have been associated with mesothelioma, CNS tumours and non-Hodgkin lymphomas (Bergsagel *et al*, 1992; Martini *et al*, 1996; Galateau-Salle *et al*, 1998; Klein *et al*, 2002; Vilchez *et al*, 2002a; Carbone *et al*, 2003; MacKenzie *et al*, 2003), even though at present there is no clear consensus on the prevalence of SV40 positivity in human tumours tissues (Klein *et al*, 2002; Carbone *et al*, 2003; Vilchez *et al*, 2003; Paracchini *et al*, 2006).

Several studies (Shah *et al*, 1974; Butel *et al*, 1999b; Li *et al*, 2002) and reviews (Kwak *et al*, 2002; Vilchez *et al*, 2002b; Vilchez *et al*, 2003; Kazory and Ducloux, 2003) have tackled the issue of the presence of polyomaviruses (BK, JC and SV40) in solid organ transplant recipients, reporting prevalence of infection between 18% (Shah *et al*, 1974) and 40% (Butel *et al*, 1999b). However, no epidemiological study has been conducted so far on cancer development in subjects who received organs from SV40 positive donors. The finding of an increase frequency of cancer in recipients from positive donors would add strength to the hypothesis of a role of SV40 in cancer aetiopathogenesis. We present here data on cancer development in transplanted subject who received organs from donors whose DNA was previously examined for the genomic insertion of SV40 (Paracchini *et al*, 2005).

## MATERIALS AND METHODS

In all, 134 solid organ donors were identified through a previous study conducted between 2002 and 2004 within the North Italian Transplant Reference Center of the Policlinico Hospital of Milano, Italy, with the aim of assessing the prevalence of SV40 genomic infection (Paracchini *et al*, 2005). None of the donors were clinically affected by cancer at the time of organ donation (Paracchini *et al*, 2005). Briefly, DNA was isolated from blood samples from organ donors, and PCR reactions were performed in order to determine the presence of SV40 sequences in the donors' DNA. Negative and positive controls were included in all sets of reactions. The pBRSV (ATCC 45019, from G Khoury) plasmid, containing the entire genome of the reference strain SV40–776, was used as the positive control in PCR amplification. Special precautions were taken in order to avoid laboratory contamination with SV40 sequences. The analysis was repeated in a blind fashion, in order to confirm the results. DNA from samples that were positive for SV40 by PCR were sequenced twice in both directions using specific primers according to the manufacturer's protocol (ABI PRISM® Big Dye TM Terminator Cycle Sequencing Ready Reaction Kit, Applied Biosystems, Foster City, CA, USA).

The organs deriving from these donors were distributed to different transplant centers in Italy (Bergamo, Bologna, Brescia, Genova, Milan, Modena, Padova, Palermo, Pavia, Pisa, Roma, Torino, Treviso, Udine, Varese, Vicenza, Verona). Informed consent for organ donation and evaluation of organ safety, as required by Italian law, was obtained from family members at the time of organ donation. The Hospital Ethical Committee approved the program of solid organ donation.

The follow-up of the recipients was performed through the National Transplant Center (NTC), part of the Superior Institute of Health. This Institute was created in March 2000 with the purpose of coordinating the activity of all the centres operating in the transplant system, and to assure the monitoring of transplant quality. The NTC conducts a yearly follow-up of the vital status of all the solid organ transplanted subjects in Italy, and among the other information, collects data on cancer incidence.

### Statistical analysis

Arithmetic means and s.d. of the demographic variables were calculated. Patients' survival was calculated using the actuarial method (Kaplan–Meier survival analysis). Each patient in the cohort was included in the analysis and counted from the date of transplant to the date of cancer development, or death or loss at follow-up.

The expected number of cancer cases in subjects who received organs from SV40 positive donors was calculated by applying cancer incidence rates observed among recipients of organs from SV40 negative donors. The Monte Carlo *χ*^2^ was used to test if a significant difference existed between observed and expected number of cancer cases. All the statistical analyses were performed using the SAS statistical package (version 8, SAS Institute Inc., Cary, NC, USA).

## RESULTS

Out of 134 proposed donors, 120 were actually utilised for organ donation. Of these, 12 (10%) were subsequently classified as positive for SV40 genomic insertion (Paracchini *et al*, 2005). None of the positive samples showed sequence homology with either JCV or BKV sequences.

The 120 utilised donors were Caucasians, 56.7% males, with an average age at organ donation of 48±16 years (range 21–78 years), and gave 408 solid organs (kidney, heart, pancreas, liver and lung) to 387 different recipients (Table 1). The average length of follow-up after transplant was 671±219 days (range 0–1085 days). Out of 387 transplant recipients, we obtained follow-up information on 364 subjects (94.1%). The missing information refer to 21 subjects from a transplant centre which preferred not to furnish follow-up data, and to two subjects whose organs were given to the European transplant organisation, and therefore where not followed-up by the NTC. Out of the 364 transplanted subjects, 11 developed cancer (3.0%): five after kidney transplant (three male subjects and two female subjects), five after liver transplant (four male subjects and one female subjects), and one after heart transplant (a male subject). The average actuarial incidence of cancer was 0.015 per year.

The 12 subjects subsequently identified as SV40 positive donors donated 43 organs to 41 recipients (Table 2). None of these recipients developed cancer during the follow-up period (average follow-up 671±266 days).

The remaining 108 donors who were subsequently identified as SV40 negative gave 365 organs to 346 recipients (Table 2). In all 11 of these recipients developed cancer during the follow-up period (667±219 days). The 11 patients who developed cancer received organs from 11 different donors (Table 2). These donors also donated other 35 organs, but none of the recipients developed cancer.

The expected number of cases among recipient of organs from positive donors was 1 per year, for a total of two expected cases during the whole follow-up period; however, no subject who received organs from SV40 positive donors developed a tumour (Monte Carlo *χ*^2^=2.05; *P*<0.001).

## DISCUSSION

Simian Virus 40 DNA infection has been detected in several but not all human tumour types (Bergsagel *et al*, 1992; Martini *et al*, 1996; Galateau-Salle *et al*, 1998; Klein *et al*, 2002; Vilchez *et al*, 2002a; Carbone *et al*, 2003; MacKenzie *et al*, 2003). The lack of consistency of the results may reflect the type of tumours examined, the age of the patients, the analytical methods used to detect genomic SV40 infection, or possible laboratory contamination (Butel and Lednicky, 1999a; Lopez-Rios *et al*, 2004; Paracchini *et al*, 2006). The summary of published studies on the association between SV40 and cancer (Butel and Lednicky, 1999a) suggests that, even if the virus may be an important co-factor in cancer development, its aetiological role is not yet proven, and the time needed for completing the carcinogenesis process is not known for SV40.

A further consideration is that SV40 DNA has been found in tumour cells, but it has also been detected in peripheral blood of otherwise healthy subjects (Paracchini *et al*, 2006). Although SV40 has been shown to be tumorigenic in animal models (Eddy *et al*, 1962), no appropriate comparable model has been developed in human subjects to study SV40 carcinogenicity. We are using a unique approach, in which the SV40 genomic status of the donors before transplant is known, and is correlated with the follow-up of the recipient for cancer development. As the information on SV40 genomic infection status of the donors was available a *priori*, it was possible to know which recipient was directly exposed to the infection after transplant.

Only a few studies have examined SV40 infection in transplanted patients (Shah *et al*, 1974; Butel *et al*, 1999b; Mylonakis *et al*, 2001), but nobody has studied cancer development in relation to SV40 infection in these patients. Among the strengths of this study is the fact that the population of transplanted patients is part of a highly controlled system of follow-up, set up at a national level, which allowed an almost complete follow-up of the subjects (over 94%). Moreover, all the donors were clinically free of cancer at the time of donation, and they were tested for the presence of SV40.

One of the limitations of the study could be the short follow-up period. In a previous study conducted in Italy on the incidence of cancer after kidney transplant, the average time to cancer development after transplant was 1200 days (Pedotti *et al*, 2003), while the average follow-up in this study is 670 days. However, Kaposi sarcoma (KS) and post transplantation lymphoproliferative disorders (PTLDs) are more likely generated by patient viral reactivation or viral transmission from the donor to recipients (Barete *et al*, 2000), and such tumours require less time to develop than solid organ tumours. Viral infection from Epstein–Barr virus (EBV) or HHV8, for example, was associated with PTLDs or KS, with incidences that were maximal during the first year (Gulley *et al*, 2003; Snanoudj *et al*, 2003; Faull *et al*, 2005) after transplant. However, since the latent period for SV40 carcinogenesis is not known, the length of follow-up necessary to assess a risk of cancer related to SV40 genomic infection in these patients remains a theoretical question. Repeated yearly follow-ups of these patients are ongoing.

This study examined the presence of genomic insertion of SV40 in transplant recipients' DNA, in order to avoid the cross reactivity to antibodies to the common human JC and BK viruses (Carter *et al*, 2003; Knowles *et al*, 2003), therefore, we have no data on carcinogenicity related to these viruses. Testing for the presence of genomic insertion of these viruses and cancer development could be valuable in future studies.

Cancer rates observed in our study are comparable to that reported in the literature in transplanted patients (Pedotti *et al*, 2003). The application of the expected cancer incidence rates (observed among recipients of organs from SV40 negative donors) to the subjects who received organs from SV40 positive donors should yield an expected number of cases around 1 per year, for a total of two expected cases during the whole follow-up period. Our data show that no subject who received organs from SV40 positive donors developed a tumour, thus the results show less than the expected incidence. Although this result does not indicate any protective effect of SV40, it is certainly consistent with a lack of carcinogenic activity of the virus in organ-transplanted subjects.

As the genomic presence of SV40 in the recipients was not tested, it could be possible that some of the subjects who developed a tumour were SV40 positive independently from the donor status. However, even if some of the recipients were SV40 positive, the fact that they did not develop any tumour during the follow-up strengthens our results. Therefore, knowing the SV40 infective status of the recipients would not decrease the strength of the result observed in the recipients of organs from positive donors, none of whom developed cancer. In conclusion, SV40 positive donors do not seem to increase the risk of cancer after transplantation.

## Figures and Tables

**Table 1:** Description of the study population

**Organ**	**No. of donors**	**No. of organs**	**No. of recipients**	**No. donors/No. of recipients**	**No. of double/combined transplants**
Kidney	115	218	216	0.53	2 double kidneys
Liver	104	113	113	0.92	2 combined with kidney
Heart	50	50	50	1.00	
Pancreas	14	14	14	1.00	13 combined with kidney
Lung	13	13	9	1.44	4 double lungs
					
Total	120	408	387	0.31	21

**Table 2:** Distribution of the organs deriving from donors tested for SV40 genomic insertion

**Type of organ**	**No. of organs**	**Recipients**	**Donors**	**Follow-up (days) mean±s.d.**	**No. of cancers**
*Donors positive for SV40*					
Kidney	24	23	12	758±184	0/23
Liver	13	13	12	577±279	0/13
Heart	5	5	5	472±72	0/5
Pancreas	1	1	1	535	0/1
Total	43	41	12	671±266	0/41
					
*Donors negative for SV40*					
Kidney	194	193	103	708±198	5/193 (2.6%)^a^
Liver	100	98	92	682±232	5/100 (5.0%)^b^
Heart	45	45	45	448±193	1/45 (2.2%)^c^
Pancreas	13	13	13	715±137	0/13
Lung	13	8	7	796±75	0/9
Total	365	346	108	667±219	11/346 (3.2%)

a Three male subjects with skin (*n*=2), and Kaposi; two female subjects with uterus and kidney.

b Four male subjects with liver recurrence (*n*=3), and lung; one female subject with liver recurrence.

c Male with lung cancer.
